# Subacute Invasive Pulmonary Aspergillosis (IPA) Is a Challenging Diagnosis

**DOI:** 10.7759/cureus.32833

**Published:** 2022-12-22

**Authors:** Marco Fernandes, Cristiana Camacho, Cláudio Gouveia, Beatriz Chambino, Ana Margarida Ribeiro

**Affiliations:** 1 Internal Medicine, Hospital de São Francisco Xavier, Lisbon, PRT

**Keywords:** pulmonary disease, prolonged corticotherapy, voriconazole, aspergillus bronchopneumonia, invasive aspergillosis

## Abstract

Aspergillus is a ubiquitous fungus whose clinical manifestations and prognosis after infection depend on the host's immune status. The disease can have an insidious course, making it a challenging diagnosis that should be considered in patients with risk factors.

We report the case of a 78-year-old man with a known history of asthma-chronic obstructive pulmonary disease (COPD) overlap syndrome and idiopathic pulmonary fibrosis on long-term therapy with high-dose oral corticosteroids, hypertension, dyslipidemia, type 2 diabetes, and hypo coagulated atrial fibrillation. He was admitted to the Emergency Department (ED) for dyspnea, productive cough, and wasting syndrome. Recent hospitalization due to pneumonia of the left upper lobe (LUE) with no agent isolation is worth mentioning, treated with levofloxacin. Due to slow improvement, he underwent bronchoscopy (BFC), which revealed friable bronchial mucosa, with isolation of Candida albicans in bronchial secretions (BS) but without evidence of neoplastic cells in the pathological anatomy (PA). He completed 14 days of itraconazole. He was discharged after partial clinical improvement. One week later, he was again admitted to a medical ward because of worsening respiratory symptoms and wasting syndrome. Laboratory findings included an elevated C-reactive protein (CRP) and sedimentation rate, hypoosmolar hyponatremia, hypoproteinemia, and hypoalbuminemia. The study of hyponatremia revealed the syndrome of inappropriate secretion of antidiuretic hormone (SIADH), and the persistence of respiratory symptoms led us to perform a chest computed tomography (CT), which revealed a subsegmental LUE atelectasis. Due to suspicion of neoplasia, he repeated BFC with isolation of Aspergillus on the bronchoalveolar lavage (BAL) and PA. Subacute invasive pulmonary aspergillosis (IPA) was assumed and voriconazole was started. However, he had an unfavorable evolution with marked cachexia and hemorrhagic shock due to lower gastrointestinal bleeding as a complication of hypocoagulation resulting in death.

Chronic exposure to corticosteroids and structural lung disease are recognized risk factors for Aspergillus infection. The presentation as a wasting syndrome associated with respiratory symptoms and SIADH raised suspicion for neoplasia, which was excluded. The PA was fundamental for the definitive diagnosis of IPA. The fatal outcome, probably attributable to late diagnosis, reinforces the importance of high clinical suspicion for Aspergillus infection in patients with risk factors.

## Introduction

Aspergillus is a ubiquitous fungus that can lead to a spectrum of clinical syndromes. The degree of immunosuppression of the host determines the clinical manifestations, course, and prognosis of the disease, even though there is an increasing concern about genetics as a predisponent factor. Most people who have contact with this microorganism do not develop lung disease.

Invasive pulmonary aspergillosis (IPA) is at one end of the spectrum, affecting patients with severe defects in immune function, such as neutropenia, hematopoietic stem cell transplant (HSCT), or solid organ transplant recipients, advanced Acquired Immunodeficiency Syndrome (AIDS), chronic granulomatous disease and patients undergoing chemotherapy or on long-term therapy with high-dose corticosteroids [1]. Sometimes, pulmonary aspergillosis can have an indolent course, affecting patients with mild or no immunocompromise but with underlying lung disease [1].

Symptoms are nonspecific and usually mimic bacterial pneumonia. Constitutional symptoms such as significant weight loss can also be present and are more frequent in chronic forms of pulmonary aspergillosis [2]. IPA diagnosis can be challenging, and the gold standard is a histological examination of lung tissue [3]. Bronchoscopy with bronchoalveolar lavage (BAL) is generally helpful for diagnosing this condition - a positive BAL for Aspergillus has a sensitivity and specificity of about 50% and 97%, respectively [4].

For many years, Amphotericin B (including the new liposome forms) has been used as first-line therapy for IPA. However, some studies have shown higher favorable response rates and survival rates with voriconazole versus amphotericin B [5]. Thus, voriconazole is now approved as an option for the initial treatment of IPA.

## Case presentation

We report the case of a 78-year-old male patient with a medical history of hypocoagulated atrial fibrillation, chronic kidney disease (basal creatinine of 1.3 mg/dL), dural arteriovenous fistula, cervical spondylotic myelopathy, asthma-COPD overlap syndrome and idiopathic pulmonary fibrosis on long term (more than 1 year) therapy with high dose oral corticosteroids (prednisolone 40mg/day).

He presented with a three-month history of wasting symptoms with severe weight loss (15kg, equivalent to 21% loss of body weight), muscle atrophy, loss of appetite, and fatigue, with no fever. He was recently hospitalized for community-acquired pneumonia with productive cough and dyspnea symptoms. Urinary antigen tests for pneumococcus and legionella were negative. Blood and sputum cultures, including for mycobacteria, were also negative. He was empirically treated with seven days of levofloxacin. Due to slow clinical improvement, a bronchoscopy (BFC) was performed, revealing a friable bronchial mucosa and isolation of Candida albicans in bronchial secretions (BS). There was no evidence of neoplastic cells on the histologic examination. He completed 14 days of itraconazole with very mild improvement. After discharge, he presented progressive clinical deterioration, being confined to bed, and six days later was admitted to the Emergency Department (ED) with worsening dyspnea and productive cough. The patient had no fever, night sweats, or chest pain. On physical examination, he was hemodynamically stable and afebrile, presented bronchospasm and respiratory failure with a peripheral oxygen saturation of 81% and respiratory rate of 28 breaths per minute, the reason why he was started on bronchodilators and supplemental oxygen. Laboratory findings revealed a normal haemogram, an elevated C-reactive protein (CRP) and sedimentation rate, normal renal function, hypoosmolar hyponatremia, hypoproteinemia, and hypoalbuminemia. Laboratory findings are shown in table 1.

**Table 1:** Laboratory findings MCV - mean cell volume, MCH - mean corpuscular hemoglobin, ODI - optical density index

Laboratory parameters	Result	Normal range
Hemoglobin (g/dL)	13.2	12.0 -15.0
MCV (fL)	85	80-96.1
MCH (pg)	31	27.3-33.7
Leukocytes (x 10^9/L)	8.2	4.0-10.0
Neutrophil (%)	70%	40-80
Platelets (x 10^9/L)	200	150-400
Sedimentation rate (mm/h)	49	<35
Urea, plasma (BUN) (mg/dL)	38	17-49
Creatinine (mg/dL)	0.52	0.5-0.9
Sodium (mmol/L)	130	136-145
Potassium (mmol/L)	4.1	3.5-5.1
Chloride (mmol/L)	101	98-107
Total proteins (g/dL)	5.0	6.4-8.3
Albumin (g/dL)	3.1	3.5-5.2
C-reactive protein (mg/dL)	10	<0.5
Serum Osmolality (mOsm/kg)	265	275-295
Urine Osmolality (mOsm/kg)	920	300-800
Urine Sodium (mEq/L)	30	<20
Serum galactomannan (ODI)	1.8	<0.5

**Figure 1:**
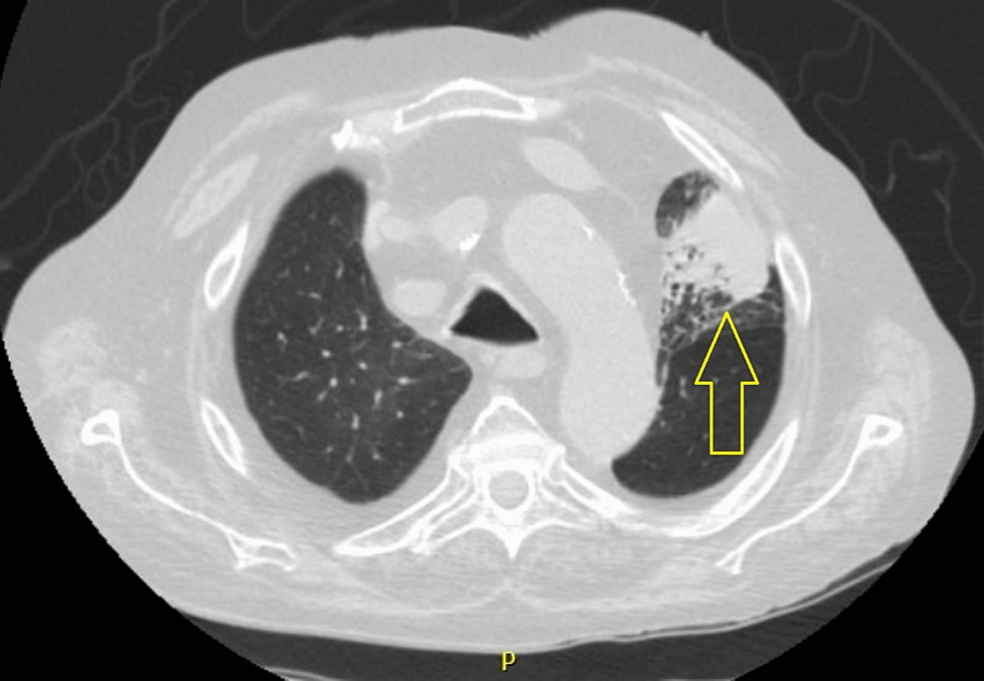
Chest CT-scan showing left upper lobe atelectasis

The chest x-ray revealed a homogeneous hypotransparency in the upper left lung field. Despite therapy with bronchodilators, he maintained significant bronchospasm with respiratory failure (O_2_ debit of five liters/minute, peripheral oxygen saturation of 95%, pO_2_ of 78mmHg), the reason why he was hospitalized. The thoracic-CT scan revealed left upper lobe atelectasis (figure 1), with no evidence of lymphadenopathies or pneumonic consolidations.

The hyponatremia study was compatible with a syndrome of inappropriate secretion of antidiuretic hormone (SIADH) - hyponatremia with low serum osmolality on a euvolemic patient and increased urinary sodium and osmolality (table 1), which reinforced the need to rule out neoplasia. The case was discussed with Pneumology, and a bronchofibroscopy with biopsy was performed - no endobronchial lesions were found, and the bronchoalveolar lavage (BAL) isolated Aspergillus. The PA examination also identified this microorganism. Additionally, the patient tested positive for serum Galactomannan, and the Interferon-Gamma Release Assay (IGRA) was negative.

To rule out neoplasia, a Positron Emission tomography (PET) scan was performed, which revealed hypermetabolism at the left superior lobe, suspicious of malignancy but unable to exclude an inflammatory/infectious etiology. A diagnosis of IPA was admitted, and the patient was started on voriconazole (6mg/kg bid on the first day, followed by 4 mg/kg/day) with dyspnea resolution, improvement of bronchial secretions, and reduction of the need for supplementary oxygen at day seven. One week later, the patient developed gastrointestinal bleeding due to hypocoagulation with enoxaparin, culminating in hemorrhagic shock and death.

## Discussion

Invasive pulmonary Aspergillosis (IPA) was first described in 1953 [6], with an increasing incidence due to the widespread use of immunosuppressive agents [7]. The major risk factor for IPA is immunodeficiency which includes HSCT and solid-organ transplantation, neutropenia, advanced AIDS, hematological malignancy, and prolonged therapy with high-dose corticosteroids [1]. The mortality rate is high, reaching 90% in hematopoietic stem-cell transplantation (HSCT) recipients [8].

Corticosteroids influence the host response against Aspergillus in several ways, such as through neutrophil function inhibition, cytokine release from type 2 T helper cells, and type 1 T helper cell suppression. In the presented case, the patient was on long-term high-dose corticosteroid therapy (40mg/day), which gave him a significant risk factor for IPA development. Although uncommon, IPA has recently been described in patients with COPD [9]. Thus, underlying lung disease is likely a risk factor for IPA. In this case, the patient had pulmonary fibrosis and asthma-COPD overlap syndrome, which probably made him more likely to develop IPA.

IPA symptoms are nonspecific and can mimic bacterial pneumonia. Lack of respiratory symptoms improvement or fever unresponsive to antibiotics presented by an immunocompromised patient should raise suspicion of this diagnosis; however, in this case, the patient never experienced fever, which the subacute evolution of the disease can explain. The patient had recently been hospitalized with community-acquired pneumonia without symptom resolution after an empirical course of levofloxacin and itraconazole, which should have raised the suspicion of an unusual microorganism like fungus, given the immunosuppressed status of the patient. Besides respiratory symptoms, the patient also presented consumption symptoms, which are more frequent in subacute/chronic pulmonary aspergillosis [2].

Besides its association with neoplasia, SIADH can also be caused by infectious diseases, namely pulmonary aspergillosis and tuberculosis. However, there is not much data on the prevalence of this association with IPA. In the reported case, the presentation with SIADH and wasting syndrome raised the suspicion of neoplasia, which was fundamental to exclude. Given its high prevalence in Portugal and the patient's respiratory symptoms, wasting syndrome, and SIADH, it was imperative to investigate tuberculosis. Besides the IGRA being negative, all the blood and BAL cultures were negative for mycobacteria. Also, PA did not identify this microorganism.

Early diagnosis of IPA remains challenging, particularly in severely immunocompromised patients. Histologic examination of lung tissue obtained by thoracoscopic or open-lung biopsy remains the gold standard for diagnosing this condition [3]. It also allows the exclusion of other diagnoses, such as malignancy, which was important in this case, given the wasting symptoms presented by the patient.

In immunocompetent patients, isolation of Aspergillus spp. in sputum samples usually has no clinical significance, representing colonization in most cases and not requiring directed therapy. However, the isolation of an Aspergillus species from sputum is highly predictive of invasive disease in immunocompromised patients. Blood cultures are usually negative in patients with confirmed IPA, as in this case [10,11].

Although neither of them is sensitive or pathognomic of IPA, typical chest CT scans findings include multiple nodules with ground-glass appearance, consolidations, the halo sign (solid nodule surrounded by a halo of ground-glass attenuation which represents hemorrhage), and the air crescent sign (peripheral crescentic collection of air surrounding a necrotic central focus of infection) [12]. The patient's chest CT scan revealed left upper lobe atelectasis with no other pathological findings besides the features of the known pulmonary chronic disease (figure 1), which makes it an unusual presentation.

Bronchoscopy with bronchoalveolar lavage (BAL) can have a crucial role in diagnosing IPA, particularly in patients with diffuse lung involvement, which was not the case. The sensitivity and specificity of a positive result of BAL fluid are about 50% and 97% [4], respectively. The patient had Aspergillus isolation from BAL, which was essential for the IPA diagnosis in this case.

Recently, there has been increasing research on the detection of Aspergillus antigens in body fluids, mainly Galactomannan (GM), and its role in IPA diagnosis. Detection of GM in body fluids like BAL, peritoneal fluid, or cerebrospinal fluid has shown significant results in recent studies [13]. In the presented clinical case, this test was not performed in BAL, which could have been helpful for the diagnosis. On the other hand, the patient was tested for serum galactomannan, which was positive, reinforcing the diagnosis.

The European Organization for Research and Treatment of Cancer and Mycoses Study Group (EORTC/MSG) criteria for diagnosing invasive pulmonary aspergillosis classified this patient as having proven IPA, given the Aspergillus identification on the pathological examination. Although several new antifungal agents have been introduced in the last years, treatment of IPA is still challenging, and mortality rates remain high, exceeding 50% in neutropenic patients and reaching 90% in HSCT recipients [8,14]. Amphotericin B was the first line of therapy for IPA for many years. However, its potentially serious side effects, like nephrotoxicity, electrolyte disturbances, and hypersensitivity, led to new lipid-based preparations like liposomal amphotericin B and lipid complex amphotericin B.

After being approved for invasive aspergillosis initial treatment, voriconazole is currently considered the treatment of choice in most patients with IPA [15]. Voriconazole was compared to amphotericin B as first-line therapy for IPA in a large prospective, randomized, multicenter trial. This study showed that patients receiving voriconazole had a higher favorable response rate at week 12 and a higher 12-week survival [5]. However, other studies showed the superiority of amphotericin B over voriconazole in specific patient groups, like cancer patients with neutropenia and patients with hematological malignancy or HSCT recipients [16,17]. The recommended voriconazole dose is 6 mg/kg bid intravenously on the first day, followed by 4 mg/kg/day. The presented patient showed significant improvement from the respiratory point of view with this dose of voriconazole. Voriconazole has a milder side-effect profile and is much better tolerated than amphotericin B. The most frequent adverse effect is visual disturbances, described as blurred vision, photophobia, and altered color perception, none of these being experienced by the patient.

Although the fatal outcome in the presented case was attributable to hemorrhagic shock due to gastrointestinal bleeding, the late IPA diagnosis increased the patient's frailty index, which probably made him more vulnerable to this nature of events. A wasting syndrome and cachexia were probably essential prognosis factors to a fatal outcome in a short-term period.

## Conclusions

IPA diagnosis remains challenging, given its broad spectrum of clinical features. In this case, the presentation as a wasting syndrome associated with respiratory symptoms and SIADH raised the suspicion of neoplasia and tuberculosis, which were less likely after the investigation. The PA was essential for the IPA's definitive diagnosis. The fatal outcome, probably attributable to late diagnosis, reinforces the importance of high clinical suspicion for Aspergillus infection in patients with risk factors.
